# Supplementary material for: Machine learning forecasts for seasonal epidemic peaks: Lessons learnt from an atypical respiratory syncytial virus season
Source: PLoS One. 2023 Sep 22;18(9):e0291932. doi: 10.1371/journal.pone.0291932 (PMC10516409; doi:10.1371/journal.pone.0291932)
Supplement: S1 File — (DOCX) [file pone.0291932.s001.docx]

# **Supplementary material**

**S1 Table 1. Mean forecast error by regression type.**

| **Regression method** | **Mean absolute forecast error** |
| --- | --- |
| Random forest | 31.6 |
| k-nearest-neighbour | 44.5 |
| Support vector machine | 53.6 |
| Linear regression | 69.1 |
| Generalised linear models with elastic net regularization (with internally optimized lambda) | 71.2 |
| Generalised linear models with elastic net regularization (without internally optimized lambda) | 78.1 |
| Extreme gradient boosting | 298.6 |

**S1 Table 2. Mean forecast error by model.**

| **regression method** | **seasonality** | **trend** | **intensity term** | | **single or 3pt average** | | **mean forecast error** |
| --- | --- | --- | --- | --- | --- | --- | --- |
| Random forest | Fourier | quadratic | linear | | 3 point | | 13.8 |
| Random forest | Fourier | quadratic | quadratic | | 3 point | | 14.3 |
| Random forest | Fourier | quadratic | quadratic | | single | | 15.2 |
| Random forest | Fourier | quadratic | linear | | single | | 15.3 |
| Random forest | Fourier | linear | quadratic | | 3 point | | 16.8 |
| Random forest | Fourier | linear | linear | | single | | 17.0 |
| Random forest | Fourier | linear | quadratic | | single | | 17.1 |
| Random forest | Fourier | linear | linear | | 3 point | | 17.6 |
| Random forest | Fourier | none | quadratic | | 3 point | | 18.3 |
| Random forest | Fourier | none | quadratic | | single | | 20.9 |
| Random forest | Fourier | none | linear | | 3 point | | 24.3 |
| Random forest | Fourier | none | linear | | single | | 25.3 |
| k-nearest-neighbour | Fourier | quadratic | linear | | single | | 25.3 |
| Random forest | none | quadratic | linear | | single | | 26.4 |
| k-nearest-neighbour | Fourier | none | linear | | single | | 26.9 |
| k-nearest-neighbour | Fourier | quadratic | linear | | 3 point | | 27.2 |
| k-nearest-neighbour | Fourier | linear | quadratic | | 3 point | | 27.3 |
| Random forest | none | quadratic | linear | | 3 point | | 27.6 |
| k-nearest-neighbour | Fourier | linear | quadratic | | single | | 27.9 |
| k-nearest-neighbour | Fourier | quadratic | quadratic | | single | | 28.5 |
| k-nearest-neighbour | Fourier | quadratic | quadratic | | 3 point | | 28.7 |
| Random forest | months | quadratic | quadratic | | single | | 29.2 |
| k-nearest-neighbour | Fourier | none | quadratic | | single | | 29.3 |
| k-nearest-neighbour | Fourier | linear | linear | | single | | 29.4 |
| Random forest | months | quadratic | linear | | 3 point | | 29.7 |
| Random forest | months | quadratic | linear | | single | | 29.8 |
| k-nearest-neighbour | Fourier | none | quadratic | | 3 point | | 29.9 |
| Random forest | months | quadratic | quadratic | | 3 point | | 30.1 |
| k-nearest-neighbour | Fourier | linear | linear | | 3 point | | 30.5 |
| k-nearest-neighbour | Fourier | none | linear | | 3 point | | 31.3 |
| Random forest | months | linear | quadratic | | 3 point | | 32.0 |
| Random forest | months | linear | linear | | single | | 33.6 |
| Random forest | none | quadratic | quadratic | | 3 point | | 34.7 |
| Random forest | months | linear | linear | | 3 point | | 34.7 |
| Random forest | none | quadratic | quadratic | | single | | 34.9 |
| Random forest | none | linear | linear | | 3 point | | 35.1 |
| Random forest | months | linear | quadratic | | single | | 36.1 |
| k-nearest-neighbour | months | linear | quadratic | | 3 point | | 36.3 |
| Random forest | none | linear | linear | | single | | 36.4 |
| Support vector machine | Fourier | none | linear | | single | | 37.2 |
| k-nearest-neighbour | months | linear | quadratic | | single | | 37.2 |
| k-nearest-neighbour | months | quadratic | quadratic | | single | | 37.4 |
| k-nearest-neighbour | months | quadratic | quadratic | | 3 point | | 37.4 |
| Support vector machine | Fourier | quadratic | quadratic | | single | | 37.4 |
| k-nearest-neighbour | months | linear | linear | | 3 point | | 37.6 |
| Support vector machine | Fourier | none | quadratic | | single | | 38.1 |
| k-nearest-neighbour | months | quadratic | linear | | 3 point | | 38.3 |
| Support vector machine | Fourier | linear | linear | | 3 point | | 38.6 |
| Support vector machine | Fourier | quadratic | quadratic | | 3 point | | 38.7 |
| Support vector machine | Fourier | linear | linear | | single | | 38.9 |
| Support vector machine | Fourier | quadratic | linear | | single | | 38.9 |
| Random forest | months | none | quadratic | | single | | 39.0 |
| Support vector machine | Fourier | quadratic | linear | | 3 point | | 39.1 |
| Support vector machine | Fourier | linear | quadratic | | 3 point | | 39.2 |
| Support vector machine | Fourier | none | quadratic | | 3 point | | 39.2 |
| k-nearest-neighbour | months | none | quadratic | | single | | 39.3 |
| Random forest | months | none | quadratic | | 3 point | | 39.6 |
| k-nearest-neighbour | months | linear | linear | | single | | 40.2 |
| Random forest | none | linear | quadratic | | single | | 40.3 |
| Support vector machine | Fourier | linear | quadratic | | single | | 40.9 |
| Support vector machine | months | quadratic | quadratic | | 3 point | | 41.4 |
| Support vector machine | Fourier | none | linear | | 3 point | | 42.5 |
| k-nearest-neighbour | months | none | linear | | 3 point | | 43.2 |
| Random forest | none | linear | quadratic | | 3 point | | 43.2 |
| k-nearest-neighbour | months | quadratic | linear | | single | | 43.4 |
| k-nearest-neighbour | months | none | quadratic | | 3 point | | 44.5 |
| Support vector machine | months | quadratic | linear | | single | | 44.7 |
| Support vector machine | months | none | quadratic | | 3 point | | 45.3 |
| k-nearest-neighbour | months | none | linear | | single | | 45.8 |
| Support vector machine | months | linear | quadratic | | single | | 46.0 |
| Support vector machine | months | linear | quadratic | | 3 point | | 46.0 |
| Support vector machine | months | linear | linear | | single | | 46.2 |
| Support vector machine | months | quadratic | linear | | 3 point | | 46.2 |
| Support vector machine | months | none | quadratic | | single | | 46.8 |
| Support vector machine | months | quadratic | quadratic | | single | | 47.7 |
| Support vector machine | months | linear | linear | | 3 point | | 48.7 |
| Support vector machine | months | none | linear | | 3 point | | 48.7 |
| Random forest | none | none | quadratic | | 3 point | | 48.9 |
| Random forest | none | none | linear | | single | | 48.9 |
| Support vector machine | months | none | linear | | single | | 49.4 |
| Random forest | none | none | linear | | 3 point | | 50.9 |
| Random forest | months | none | linear | | 3 point | | 53.4 |
| Linear regression | months | linear | linear | | 3 point | | 53.4 |
| Random forest | none | none | quadratic | | single | | 54.2 |
| Random forest | months | none | linear | | single | | 54.4 |
| Linear regression | months | quadratic | linear | | 3 point | | 55.4 |
| Linear regression | months | quadratic | quadratic | | single | | 56.0 |
| GLM with elastic net regularization (with internally optimized lambda) | months | quadratic | quadratic | single | | 56.2 | |
| Linear regression | months | none | quadratic | 3 point | | 56.5 | |
| Linear regression | months | quadratic | quadratic | 3 point | | 56.5 | |
| Linear regression | months | none | linear | 3 point | | 56.7 | |
| Linear regression | months | none | quadratic | single | | 57.3 | |
| GLM with elastic net regularization (with internally optimized lambda) | months | linear | quadratic | single | | 58.4 | |
| Linear regression | months | none | linear | single | | 58.6 | |
| GLM with elastic net regularization (with internally optimized lambda) | months | none | quadratic | single | | 58.6 | |
| Linear regression | months | linear | quadratic | single | | 58.6 | |
| GLM with elastic net regularization (with internally optimized lambda) | months | linear | linear | single | | 58.8 | |
| Linear regression | months | linear | quadratic | 3 point | | 58.8 | |
| GLM with elastic net regularization (with internally optimized lambda) | months | quadratic | quadratic | 3 point | | 59.1 | |
| k-nearest-neighbour | none | quadratic | quadratic | 3 point | | 59.3 | |
| Linear regression | months | linear | linear | single | | 59.5 | |
| Linear regression | Fourier | none | quadratic | 3 point | | 59.5 | |
| GLM with elastic net regularization (with internally optimized lambda) | Fourier | quadratic | quadratic | single | | 59.7 | |
| Linear regression | Fourier | linear | quadratic | single | | 60.3 | |
| k-nearest-neighbour | none | none | quadratic | single | | 60.5 | |
| GLM with elastic net regularization (with internally optimized lambda) | Fourier | quadratic | linear | single | | 60.5 | |
| GLM with elastic net regularization (with internally optimized lambda) | months | none | quadratic | 3 point | | 61.0 | |
| GLM with elastic net regularization (with internally optimized lambda) | months | none | linear | single | | 61.1 | |
| k-nearest-neighbour | none | quadratic | linear | 3 point | | 61.2 | |
| Linear regression | Fourier | quadratic | quadratic | 3 point | | 61.2 | |
| GLM with elastic net regularization (with internally optimized lambda) | months | none | linear | 3 point | | 61.3 | |
| GLM with elastic net regularization (with internally optimized lambda) | months | quadratic | linear | single | | 61.4 | |
| k-nearest-neighbour | none | quadratic | linear | single | | 61.5 | |
| Linear regression | Fourier | linear | quadratic | 3 point | | 61.5 | |
| GLM with elastic net regularization (with internally optimized lambda) | Fourier | none | linear | single | | 61.5 | |
| k-nearest-neighbour | none | linear | linear | 3 point | | 61.6 | |
| GLM with elastic net regularization (with internally optimized lambda) | Fourier | linear | linear | single | | 61.8 | |
| GLM with elastic net regularization (with internally optimized lambda) | Fourier | quadratic | quadratic | 3 point | | 62.3 | |
| Linear regression | Fourier | none | quadratic | single | | 62.4 | |
| Linear regression | months | quadratic | linear | single | | 62.6 | |
| Linear regression | Fourier | linear | linear | single | | 62.8 | |
| k-nearest-neighbour | none | linear | quadratic | 3 point | | 63.0 | |
| GLM with elastic net regularization (with internally optimized lambda) | Fourier | linear | quadratic | single | | 63.5 | |
| GLM with elastic net regularization (with internally optimized lambda) | Fourier | none | quadratic | single | | 63.6 | |
| Linear regression | Fourier | quadratic | linear | 3 point | | 63.7 | |
| Linear regression | Fourier | quadratic | quadratic | single | | 63.8 | |
| GLM with elastic net regularization (with internally optimized lambda) | months | linear | quadratic | 3 point | | 63.9 | |
| GLM with elastic net regularization (with internally optimized lambda) | Fourier | none | quadratic | 3 point | | 64.4 | |
| Linear regression | Fourier | none | linear | single | | 65 | |
| Linear regression | Fourier | none | linear | 3 point | | 65.1 | |
| GLM with elastic net regularization (with internally optimized lambda) | Fourier | linear | linear | 3 point | | 65.1 | |
| GLM with elastic net regularization | Fourier | quadratic | quadratic | single | | 65.1 | |
| Linear regression | Fourier | linear | linear | 3 point | | 65.3 | |
| GLM with elastic net regularization (with internally optimized lambda) | months | quadratic | linear | 3 point | | 65.4 | |
| k-nearest-neighbour | none | none | quadratic | 3 point | | 65.6 | |
| k-nearest-neighbour | none | quadratic | quadratic | single | | 65.8 | |
| Linear regression | Fourier | quadratic | linear | single | | 65.9 | |
| GLM with elastic net regularization | Fourier | none | linear | single | | 66.0 | |
| GLM with elastic net regularization (with internally optimized lambda) | Fourier | quadratic | linear | 3 point | | 66.2 | |
| GLM with elastic net regularization | Fourier | quadratic | linear | single | | 67.6 | |
| GLM with elastic net regularization | Fourier | linear | linear | single | | 67.7 | |
| GLM with elastic net regularization | Fourier | quadratic | quadratic | 3 point | | 67.8 | |
| GLM with elastic net regularization (with internally optimized lambda) | Fourier | linear | quadratic | 3 point | | 67.9 | |
| GLM with elastic net regularization (with internally optimized lambda) | months | linear | linear | 3 point | | 68.3 | |
| GLM with elastic net regularization | months | quadratic | linear | single | | 68.8 | |
| GLM with elastic net regularization | months | none | linear | single | | 68.9 | |
| GLM with elastic net regularization | Fourier | quadratic | linear | 3 point | | 69.2 | |
| GLM with elastic net regularization | Fourier | linear | quadratic | single | | 69.8 | |
| k-nearest-neighbour | none | none | linear | 3 point | | 69.8 | |
| GLM with elastic net regularization | Fourier | none | quadratic | 3 point | | 69.9 | |
| GLM with elastic net regularization (with internally optimized lambda) | Fourier | none | linear | 3 point | | 69.9 | |
| GLM with elastic net regularization | Fourier | linear | linear | 3 point | | 70.0 | |
| GLM with elastic net regularization | Fourier | linear | quadratic | 3 point | | 70.1 | |
| k-nearest-neighbour | none | linear | linear | single | | 70.4 | |
| k-nearest-neighbour | none | linear | quadratic | single | | 70.5 | |
| GLM with elastic net regularization | months | linear | linear | single | | 70.6 | |
| GLM with elastic net regularization | Fourier | none | linear | 3 point | | 70.6 | |
| Support vector machine | none | none | linear | single | | 70.9 | |
| k-nearest-neighbour | none | none | linear | single | | 70.9 | |
| GLM with elastic net regularization | months | quadratic | quadratic | single | | 71.0 | |
| Support vector machine | none | linear | quadratic | single | | 71.2 | |
| GLM with elastic net regularization | months | linear | quadratic | single | | 71.3 | |
| Support vector machine | none | quadratic | quadratic | 3 point | | 71.4 | |
| GLM with elastic net regularization | Fourier | none | quadratic | single | | 71.6 | |
| GLM with elastic net regularization | months | linear | quadratic | 3 point | | 71.7 | |
| GLM with elastic net regularization | months | quadratic | quadratic | 3 point | | 73.0 | |
| GLM with elastic net regularization | months | none | quadratic | 3 point | | 73.2 | |
| Support vector machine | none | linear | linear | 3 point | | 73.5 | |
| Support vector machine | none | quadratic | linear | 3 point | | 73.8 | |
| GLM with elastic net regularization | months | none | quadratic | single | | 74.3 | |
| GLM with elastic net regularization | months | quadratic | linear | 3 point | | 74.4 | |
| GLM with elastic net regularization | months | none | linear | 3 point | | 74.5 | |
| Support vector machine | none | none | quadratic | single | | 74.9 | |
| Support vector machine | none | quadratic | quadratic | single | | 75.9 | |
| GLM with elastic net regularization | months | linear | linear | 3 point | | 76.6 | |
| Support vector machine | none | none | quadratic | 3 point | | 77.1 | |
| Support vector machine | none | linear | quadratic | 3 point | | 77.4 | |
| Support vector machine | none | linear | linear | single | | 77.4 | |
| Support vector machine | none | quadratic | linear | single | | 77.6 | |
| Linear regression | none | none | quadratic | 3 point | | 79.5 | |
| Support vector machine | none | none | linear | 3 point | | 82.8 | |
| Linear regression | none | none | linear | single | | 82.9 | |
| GLM with elastic net regularization (with internally optimized lambda) | none | linear | quadratic | 3 point | | 84.0 | |
| GLM with elastic net regularization (with internally optimized lambda) | none | quadratic | quadratic | 3 point | | 84.0 | |
| Linear regression | none | linear | quadratic | single | | 84.1 | |
| Linear regression | none | none | quadratic | single | | 84.5 | |
| GLM with elastic net regularization (with internally optimized lambda) | none | linear | quadratic | single | | 84.9 | |
| GLM with elastic net regularization (with internally optimized lambda) | none | quadratic | quadratic | single | | 85.2 | |
| Linear regression | none | linear | quadratic | 3 point | | 85.3 | |
| Linear regression | none | quadratic | linear | 3 point | | 85.9 | |
| GLM with elastic net regularization (with internally optimized lambda) | none | none | quadratic | single | | 86.8 | |
| GLM with elastic net regularization | none | quadratic | linear | 3 point | | 87.0 | |
| Linear regression | none | quadratic | quadratic | single | | 87.3 | |
| Linear regression | none | quadratic | quadratic | 3 point | | 87.5 | |
| GLM with elastic net regularization (with internally optimized lambda) | none | none | quadratic | 3 point | | 87.9 | |
| Linear regression | none | quadratic | linear | single | | 88.4 | |
| Linear regression | none | linear | linear | single | | 89.3 | |
| Linear regression | none | none | linear | 3 point | | 89.9 | |
| GLM with elastic net regularization (with internally optimized lambda) | none | quadratic | linear | 3 point | | 90.2 | |
| GLM with elastic net regularization (with internally optimized lambda) | none | none | linear | 3 point | | 90.5 | |
| GLM with elastic net regularization (with internally optimized lambda) | none | linear | linear | single | | 90.9 | |
| GLM with elastic net regularization | none | none | quadratic | 3 point | | 91.0 | |
| GLM with elastic net regularization (with internally optimized lambda) | none | none | linear | single | | 91.2 | |
| GLM with elastic net regularization (with internally optimized lambda) | none | quadratic | linear | single | | 91.5 | |
| GLM with elastic net regularization | none | none | linear | 3 point | | 91.9 | |
| GLM with elastic net regularization | none | quadratic | linear | single | | 92.3 | |
| GLM with elastic net regularization | none | linear | quadratic | single | | 92.5 | |
| GLM with elastic net regularization | none | quadratic | quadratic | 3 point | | 92.8 | |
| GLM with elastic net regularization | none | linear | linear | single | | 92.9 | |
| GLM with elastic net regularization | none | linear | quadratic | 3 point | | 93.4 | |
| GLM with elastic net regularization | none | none | quadratic | single | | 94.3 | |
| Linear regression | none | linear | linear | 3 point | | 94.9 | |
| GLM with elastic net regularization | none | quadratic | quadratic | single | | 95.3 | |
| GLM with elastic net regularization | none | none | linear | single | | 97.1 | |
| GLM with elastic net regularization (with internally optimized lambda) | none | linear | linear | | 3 point | | 97.6 |
| GLM with elastic net regularization | none | linear | linear | | 3 point | | 98.1 |
| Extreme gradient boosting | months | quadratic | quadratic | | 3 point | | 279.9 |
| Extreme gradient boosting | months | linear | quadratic | | single | | 286.8 |
| Extreme gradient boosting | Fourier | quadratic | linear | | 3 point | | 291.7 |
| Extreme gradient boosting | Fourier | linear | linear | | single | | 292.9 |
| Extreme gradient boosting | months | quadratic | linear | | single | | 293.2 |
| Extreme gradient boosting | Fourier | none | linear | | single | | 293.5 |
| Extreme gradient boosting | months | none | quadratic | | 3 point | | 293.6 |
| Extreme gradient boosting | months | none | quadratic | | single | | 294.0 |
| Extreme gradient boosting | Fourier | quadratic | linear | | single | | 294.9 |
| Extreme gradient boosting | months | quadratic | linear | | 3 point | | 295.0 |
| Extreme gradient boosting | months | quadratic | quadratic | | single | | 295.4 |
| Extreme gradient boosting | months | linear | quadratic | | 3 point | | 295.5 |
| Extreme gradient boosting | Fourier | none | quadratic | | 3 point | | 296.4 |
| Extreme gradient boosting | months | linear | linear | | 3 point | | 296.5 |
| Extreme gradient boosting | none | quadratic | quadratic | | 3 point | | 296.9 |
| Extreme gradient boosting | none | quadratic | quadratic | | single | | 297.0 |
| Extreme gradient boosting | Fourier | linear | quadratic | | single | | 297.5 |
| Extreme gradient boosting | Fourier | linear | linear | | 3 point | | 297.7 |
| Extreme gradient boosting | none | linear | quadratic | | 3 point | | 298.0 |
| Extreme gradient boosting | Fourier | none | linear | | 3 point | | 298.4 |
| Extreme gradient boosting | none | linear | linear | | single | | 299.0 |
| Extreme gradient boosting | months | none | linear | | single | | 299.9 |
| Extreme gradient boosting | Fourier | none | quadratic | | single | | 299.9 |
| Extreme gradient boosting | Fourier | linear | quadratic | | 3 point | | 300.2 |
| Extreme gradient boosting | months | none | linear | | 3 point | | 300.5 |
| Extreme gradient boosting | none | quadratic | linear | | 3 point | | 300.9 |
| Extreme gradient boosting | none | none | linear | | 3 point | | 301.4 |
| Extreme gradient boosting | none | linear | linear | | 3 point | | 302.0 |
| Extreme gradient boosting | Fourier | quadratic | quadratic | | 3 point | | 302.4 |
| Extreme gradient boosting | months | linear | linear | | single | | 306.2 |
| Extreme gradient boosting | none | none | quadratic | | single | | 307.4 |
| Extreme gradient boosting | none | quadratic | linear | | single | | 307.5 |
| Extreme gradient boosting | Fourier | quadratic | quadratic | | single | | 308.3 |
| Extreme gradient boosting | none | linear | quadratic | | single | | 308.3 |
| Extreme gradient boosting | none | none | quadratic | | 3 point | | 310.5 |
| Extreme gradient boosting | none | none | linear | | single | | 311.8 |

**S1 Table 3. Models with lowest mean forecast error by forecast lead time. All have a quadratic trend.**

| Forecast lead | Mean forecast error | Regression method | seasonality | quadratic | single |
| --- | --- | --- | --- | --- | --- |
| 1 | 6.8 | Linear regression | months | linear | single |
| 2 | 10.0 | Random forest | Fourier | quadratic | three_points |
| 3 | 11.6 |  |  | linear | three_points |
| 4 | 12.0 |  |  | quadratic | three_points |
| 5 | 12.6 |  |  | quadratic | three_points |
| 6 | 12.8 |  |  | quadratic | three_points |
| 7 | 13.4 |  |  | quadratic | three_points |
| 8 | 15.1 |  |  | quadratic | three_points |
| 9 | 14.3 |  |  | quadratic | three_points |
| 10 | 14.4 |  |  | linear | three_points |
| 11 | 13.0 |  |  | linear | three_points |
| 12 | 13.1 |  |  | linear | three_points |
| 13 | 13.9 |  |  | linear | three_points |
| 14 | 13.7 |  |  | linear | three_points |
| 15 | 13.8 |  |  | linear | three_points |
| 16 | 13.5 |  |  | linear | three_points |
| 17 | 13.5 |  |  | linear | three_points |
| 18 | 14.2 |  |  | linear | three_points |
| 19 | 13.4 |  |  | linear | three_points |
| 20 | 13.8 |  |  | linear | three_points |
| 21 | 14.1 |  |  | linear | three_points |
| 22 | 13.7 |  |  | linear | three_points |
| 23 | 14.2 |  |  | linear | three_points |
| 24 | 13.9 |  |  | linear | three_points |
| 25 | 15.3 |  |  | linear | three_points |
| 26 | 14.9 |  |  | quadratic | three_points |
| 27 | 14.9 |  |  | linear | three_points |
| 28 | 14.9 |  |  | quadratic | single |
